# Risk of squamous cell skin cancer after organ transplant associated with antibodies to cutaneous papillomaviruses, polyomaviruses, and *TMC6/8 (EVER1/2)* variants

**DOI:** 10.1002/cam4.280

**Published:** 2014-06-10

**Authors:** Margaret M Madeleine, Joseph J Carter, Lisa G Johnson, Gregory C Wipf, Connie Davis, Daniel Berg, Karen Nelson, Janet R Daling, Stephen M Schwartz, Denise A Galloway

**Affiliations:** 1 Program in Epidemiology, Fred Hutchinson Cancer Research CenterSeattle, Washington; 2 Department of Epidemiology, University of WashingtonSeattle, Washington; 3 Human Biology Division, Fred Hutchinson Cancer Research CenterSeattle, Washington; 4 Division of Nephrology, University of WashingtonSeattle, Washington; 5 Division of Dermatology, University of WashingtonSeattle, Washington; 6 Immunogenetics Laboratory, Puget Sound Blood CenterSeattle, Washington

**Keywords:** Cutaneous human papillomavirus, epidemiology, organ transplant, polyomavirus, squamous cell skin cancer

## Abstract

Squamous cell skin cancer (SCSC) disproportionately affects organ transplant recipients, and may be related to increased viral replication in the setting of immune suppression. We conducted a nested case–control study among transplant recipients to determine whether SCSC is associated with antibodies to cutaneous human papillomaviruses (HPV), to genes associated with a rare genetic susceptibility to HPV (*TMC6/TMC8*), or to human polyomaviruses (HPyV). Cases (*n* = 149) had histologically confirmed SCSC, and controls (*n* = 290) were individually matched to cases on time since transplant, type of transplant, gender, and race. All subjects had serum drawn immediately prior to transplant surgery. Antibodies to 25 cutaneous HPVs and six HPyVs were assayed by detection of binding to virus-like particles, and 11 TMC6/8 variants were genotyped. After correction for multiple comparisons, only antibodies to HPV37 were associated with SCSC (OR 2.0, 95% CI 1.2–3.4). Common genetic variants of TMC6/8 were not associated with SCSC, but three variants in *TMC8* (rs12452890, rs412611, and rs7208422) were associated with greater seropositivity for species 2 betapapillomaviruses among controls. This study suggests that some betaHPVs, but not polyomaviruses, may play a role in the excess risk of SCSC among transplant recipients.

## Introduction

Most solid organ transplant recipients take maintenance immunosuppressant medications to prevent graft rejection, and therefore, have increased susceptibility to viral infections and excess risk of virus-related cancers. A large registry-based study of transplant recipients and cancer risk 1 reported highly elevated standardized incidence ratios (SIRs) associated with virus-related cancers. Examples include Kaposi sarcoma, linked to human herpesvirus type 8 infection (SIR 61), liver cancer often caused by hepatitis B or C viruses (SIR 11), non-Hodgkin lymphoma, associated with Epstein–Barr virus (SIR 11). In contrast, cancers not associated with viruses, such as breast and prostate cancer, do not occur in excess among transplant recipients 1.

The most commonly occurring malignancy posttransplant is nonmelanoma skin cancer, and immunosuppressive medications and prior sun exposure are important in their etiology 2,3. Although prior studies have reported very high, >50-fold, increased risks for squamous cell skin cancer (SCSC) among transplant recipients compared to the general population 4,5, no definitive viral etiology has been established for SCSC 6. Transplant physicians and dermatologists prioritize prevention and early detection efforts to avoid SCSC that occurs in excess after transplant. Even so, some transplant recipients develop aggressive SCSC with early onset, multiple tumors, and high recurrence rates 7,8.

Specific types of SCSC have been definitively associated with two viruses, human papillomaviruses (HPV) and human polyomaviruses (HPyV). Cutaneous HPV types were initially associated with SCSC because genus betapapillomavirus (betaHPV) were detected in skin tumors from individuals with a rare genetic disorder 9,10 attributed to mutations in the *TMC6*/*8* (*EVER1/2*) genes 11. The TMC6/8 proteins may act as barriers to HPV transcription factors and replication, with their loss leading to susceptibility to HPV-related skin lesions 12. However, no high-risk HPV type or subset of types has been consistently associated with SCSC in transplant recipients 13–15.

The Merkel cell polyomavirus 16 (MCPyV) is strongly associated with a rare cutaneous cancer, Merkel cell carcinoma 17. There are six cutaneous human polyomaviruses (HPyV: MCPyV, JCPyV, KIPyV, WUPyV, HPyV6, and HPyV7) that have cutaneous pathogenic potential 18. Recent studies provided mixed results concerning the possibility that HPyV 18–20 or HPV 13,21 is associated with SCSC among transplant recipients. In this study, we investigated whether antibodies to cutaneous HPyV or HPV, or genetic variation in TMC6/8, were associated with SCSC among highly susceptible transplant recipients.

## Materials and Methods

### Study population

This nested case–control study included participants in the Skin Cancer after Organ Transplant (SCOT) cohort study 22. The SCOT cohort includes ∼2000 renal and cardiac transplant recipients who received their transplant in the Seattle area between 1995 and 2010. Participants resided in Washington, Wyoming, Alaska, Montana, or Idaho at the time of transplant. Exclusions included history of transplant prior to 1995, age less than 18 years old at time of transplant, inability to communicate in English, a history of SCSC prior to transplant, and graft failure within 3 months of transplant. Study procedures were approved by the Institutional Review Board of the Fred Hutchinson Cancer Research Center.

Participants were asked to report any skin condition that required a biopsy, and biopsy reports were reviewed to confirm a diagnosis of invasive SCSC. The first instance of pathology-confirmed SCSC after transplant served as the case diagnosis date. Controls were participants without SCSC, matched to cases on year of transplant (±2 years), age at transplant (±5 years), time since transplant (exact months), transplant hospital, donor type (deceased or living), organ transplanted (kidney, kidney and pancreas, or heart), gender, and race (white or not white).

We enrolled 172 cases (87% of 198 confirmed SCSC by pathology report review) and 332 controls (80% of the 415 matched controls selected from the cohort) in the nested case–control study. There were eight participants initially identified as controls based on a prior mailed survey, who reported SCSC at the time of the in-person interview. These participants became cases for whom new controls were selected.

### Interview

We collected detailed information during an in-person interview. In addition to history of biopsies and dermatologic visits, we asked questions about sun exposure history included skin type, amount of time spent outdoors, susceptibility to burning and tanning, use of tanning beds, and sun avoidance practices. We also asked about medical history, including type of immunosuppressive therapies, residence history, and ethnicity.

### Biospecimens

Pretransplant serum samples were collected and stored at the time of transplant by the Puget Sound Blood Center. We retrieved and tested serum samples from 149 cases (86.6%) and 290 controls (87.3%). In addition, 90% of cases and 84% of controls agreed to a blood draw or saliva sample (OraGene; DNAGenotek, Kanata, ON, Canada) collection at the time of the interview, which served as the source of genomic DNA. There was an overlap in available serology data and DNA results data for 143 cases and 259 controls.

### Serologic assays

The antibody detection method was based on glutathione-S-transferase (GST) capture enzyme-linked immunosorbent assay (ELISA) described by Sehr et al. 23. in combination with fluorescent bead technology (Luminex, Austin, TX) 24. The antibody bound to viral antigen was quantified via the median fluorescence intensity (MFI) of at least 100 beads of the same internal color on the Luminex platform.

HPV L1 DNA types that were cloned into the expression plasmids were received as gifts from the following colleagues: types 12, 14, 21, and 22 from G; Orth (Paris, France), types 37 and 47 from E. M. de Villiers (Heidelberg, Germany); and HPV type 96 from O. Forslund (Malmö, Sweden). The other HPVL1 expression plasmids were from M. Pawlita (Heidelberg, Germany), who also provided plasmids for BKPyV and JCPyV. All L1 proteins were partially purified by ion exchange chromatography 25, which improved reactivity. HPyV6 and HPyV7 sequences were purchased from Addgene (Cambridge, MA). Cloning of MCPyV, WUPyV, and KIPyV plasmids was previously described 26.

Blood samples from cases and controls were tested for antibodies to 25 betaHPVs and six polyomaviruses. Laboratory personnel were blinded, and case and control samples were randomly ordered. Concordance of 10% replicates for the HPV panel, ranged from 0.70 to 0.96, and for the HPyV replicates ranged from 0.79 to 0.92. The cut point for a seropositive result was set at MFI > 500 for HPV and MFI > 5000 for HPyV antigens.

### Genetic assay

Single-nucleotide polymorphisms (SNPs) were selected from the *TMC6* and *TMC8* region (ch 17: 76,108,999–76,139,049), plus 4000 base pairs 5′ and 3′ of the region, to maximally capture common genetic variation 27. Selected SNPs (*n* = 12) had a minor allele frequency of at least 0.05% among Caucasians in the reference population (HapMap), and were genotyped using the VeraCode platform. One SNP from *TMC8* (rs17773854) was excluded from analysis, due to call proportion <0.90 and HWE *P* < 0.01. Approximately 7% quality control replicates were included in the assay. Replicate concordance across SNPs ranged from 94.1% to 100%, averaging 99.1%.

### Statistical analysis

Odds ratios (OR) and 95% confidence intervals (CI) were generated by conditional logistic regression models. Further adjustment for age at reference (i.e., age at cancer diagnosis for cases and age at a comparable time point posttransplant for matched controls) was added to reduce possible residual confounding. History of UV exposure (susceptibility to burning or tanning and number of sunburns) or type of immunosuppressive drug (history of ever use of each of the following: azathioprine, cyclosporine, tacrolimus, mycophenolate mofetil) did not appreciably change the estimates and were not included in final estimates. To correct for multiple comparisons, we used a Holm–Bonferroni step-down procedure 28 to determine statistically significant estimates (corrected *P* < 0.05) for individual betaHPV types. Associations between variants and SCSC were computed assuming a log-additive conditional model.

## Results

The distribution of matching factors for this nested case–control study is detailed in Table S1. Serum samples were collected immediately prior to transplant, and cases were diagnosed for SCSC with an average of 5.4 years (3.2 SD) after transplant. The majority of study participants were seropositive for HPyV6, KIPyV, MCPyV, and WUPyV (Table 1). The JCPyV assay had lower levels of positivity and the WUPyV had highest levels. There was no association between risk of SCSC and seropositivity to individual HPyV or grouped betaHPV types overall, multiple betaHPV types, or betaHPV phylogenetic species.

**Table 1:** Risk of squamous cell skin cancer among solid organ transplant recipients associated with antibodies to cutaneous viruses, Seattle area SCOT cohort 1995–2010

	Controls (*n* = 290)		Cases (*n* = 149)			
				
	*n*	(%)	*n*	(%)	OR	(95% CI)
Polyomaviruses^1^						
JCPyV	47	22.7	16	14.5	0.8	(0.4–1.6)
KIPyV	146	70.5	78	70.9	1.1	(0.6–2.0)
MCPyV	131	63.3	63	57.3	0.7	(0.4–1.2)
WUPyV	172	83.1	99	90.0	1.2	(0.6–2.6)
HPyV6	137	60.1	65	52.0	0.6	(0.4–1.0)
HPyV7	85	37.3	57	45.6	1.2	(0.7–1.9)
BetaHPVs Types						
Seronegative	73	(25.2)	41	(27.5)	ref	
Seropositive	217	(74.8)	108	(72.5)	1.0	(0.6–1.5)
1 type	61	(21.0)	34	(22.8)	1.0	(0.6–1.8)
2 to 4 types	90	(31.0)	31	(20.8)	0.6	(0.3–1.1)
5 to 9 types	34	(11.7)	24	(16.1)	1.5	(0.7–3.0)
10+ types	32	(11.0)	19	(12.8)	1.2	(0.6–2.6)
Species 1^2^						
Negative	129	(44.5)	74	(49.7)	ref	
Positive	161	(55.5)	75	(50.3)	0.8	(0.5–1.3)
Species 2						
Negative	144	(49.7)	73	(49.0)	ref	
Positive	146	(50.3)	76	(51.0)	1.1	(0.7–1.7)
Species 3						
Negative	222	(76.6)	114	(76.5)	ref	
Positive	68	(23.4)	35	(23.5)	1.1	(0.7–1.8)

CI, confidence intervals; OR, odds ratios; SCOT, Skin Cancer after Organ Transplant.

1 Polyomavirus serology performed on a subset of participants (*N* = 110 cases and *N* = 207 controls).

2 Genus and species grouped according to phylogenetic description of de Villiers et al. 56.

Individual results for the HPV assays and risk of SCSC are shown in Table 2. Among the betaHPV types assayed, antibodies to HPV37 occurred most commonly in those with SCSC (26.8% in cases vs. 17.6% in controls), and were associated with a significantly increased risk of SCSC (OR 2.0, 95% CI 1.2–3.4, *P* = 0.005). Elevated but nonsignificantly increased risks were associated with HPV15, HPV20, and HPV36. There was also an increased risk of SCSC associated with HPV1 (OR 1.9, 95% CI 1.1–3.1, *P* = 0.042), the common plantar wart virus.

**Table 2:** Risk of squamous cell skin cancer among transplant recipients associated with HPV seropositivity, Seattle area SCOT cohort 1995–2010

		Controls (*n* = 290)		Cases (*n* = 149)				
						
HPV type	Genus/Species^1^	*n*	(%)	*n*	(%)	OR	(95% CI)	*P*^2^
HPV1	*μ*	137	(47.2)	82	(55.0)	1.6	(1.0–2.5)	0.042
HPV2	*α*	47	(16.2)	29	(19.5)	1.4	(0.8–2.5)	
HPV4	*γ*	156	(53.8)	79	(53.0)	1.1	(0.7–1.6)	
HPV8	*β*1	54	(18.6)	27	(18.1)	1.0	(0.6–1.7)	
HPV9	*β*2	71	(24.5)	32	(21.5)	0.8	(0.5–1.4)	
HPV12	*β*1	63	(21.7)	36	(24.2)	1.2	(0.7–2.1)	
HPV14	*β*1	46	(15.9)	26	(17.4)	1.2	(0.7–2.3)	
HPV15	*β*2	44	(15.2)	30	(20.1)	1.6	(0.9–2.8)	
HPV17	*β*2	44	(15.2)	24	(16.1)	1.3	(0.7–2.3)	
HPV20	*β*1	45	(15.5)	32	(21.5)	1.6	(0.9–2.9)	
HPV21	*β*1	50	(17.2)	30	(20.1)	1.2	(0.7–2.1)	
HPV22	*β*2	30	(10.3)	16	(10.7)	1.3	(0.6–2.4)	
HPV23	*β*2	27	(9.3)	14	(9.4)	1.2	(0.6–2.4)	
HPV24	*β*1	52	(17.9)	22	(14.8)	1.0	(0.5–1.8)	
HPV36	*β*1	37	(12.8)	26	(17.4)	1.6	(0.9–2.8)	
HPV37	*β*2	51	(17.6)	40	(26.8)	2.0	(1.2–3.4)	0.005
HPV38	*β*2	31	(10.7)	12	(8.1)	0.8	(0.4–1.9)	
HPV47	*β*1	42	(14.5)	28	(18.8)	1.3	(0.8–2.2)	
HPV49	*β*3	39	(13.4)	27	(18.1)	1.3	(0.8–2.3)	
HPV75	*β*3	41	(14.1)	23	(15.4)	1.3	(0.7–2.3)	
HPV76	*β*3	32	(11.0)	18	(12.1)	1.0	(0.5–1.9)	
HPV80	*β*2	34	(11.7)	23	(15.4)	1.3	(0.7–2.3)	
HPV92	*β*4	23	(7.9)	15	(10.1)	1.4	(0.7–2.9)	
HPV93	*β*1	7	(2.4)	5	(3.4)	1.5	(0.4–5.5)	
HPV96	*β*5	41	(14.1)	24	(16.1)	1.2	(0.7–2.2)	

CI, confidence intervals; HPV, human papillomaviruses; OR, odds ratios; SCOT, Skin Cancer after Organ Transplant.

1 Species grouped according to phylogenetic description of de Villiers et al. 56.

2 *P*-values are corrected for multiple comparisons.

In genetic analyses, we explored whether common variants in *TMC6*/*8* were related to betaHPV seropositivity (Table 3). Among controls, three variants in *TMC8* were associated with greater seropositivity for antibodies against betaHPV species 2: the G allele of rs12452890; the A allele of rs412611; and the A allele of rs7208422. We then assessed whether variant alleles in the TMC6/8 region were associated with risk of SCSC (Table S2), and found no increased risk of SCSC associated with these variants. There were suggestive, but not significant associations with two SNPs in TMC6 and reduced risk of SCSC (G allele of rs16970842 with OR 0.56, 95% CI 0.29–1.08, and G allele of rs7218589 with OR 0.53, 95% CI 0.27–1.04).

**Table 3:** Proportion of transplant recipients controls seropositive for betaHPV species^2^ 1–3 by *TMC6/8* variants, Seattle area SCOT cohort 1995–2010

	Species 1 (*N* = 141)		Species 2 (*N* = 129)		Species 3 (*N* = 61)	
			
Variant	*n*	%	*n*	%	*n*	%
rs12452890						
AA	31	50.8	24	39.3	14	22.9
AG	76	56.7	63	47.0	32	23.9
GG	34	53.1	42	65.6^1^	15	23.4
rs16970842						
AA	113	53.8	105	50.0	53	25.2
AG/GG	28	57.1	24	49.0	8	16.3
rs16970849						
GG	130	54.9	123	51.9	58	24.5
GA/AA	11	50.0	6	27.3	3	13.6
rs2871647						
AA	109	51.2	101	47.4	48	22.5
AC/CC	32	69.6^1^	28	62.2	13	28.3
rs383603						
CC	81	52.9	78	51.0	33	21.6
CG	49	55.1	44	49.4	24	27.0
GG	11	64.7	7	41.2	4	23.5
rs412611						
GG	120	54.1	103	46.4	51	23.0
GA	21	56.8	26	70.3^1^	10	27.0
rs454138						
GG	54	56.8	50	52.6	27	28.4
GC	65	53.3	53	43.4	24	19.7
CC	22	52.4	26	61.9	10	23.8
rs7208422						
AA	39	59.1	41	62.1	15	23.4
AT	70	54.3	63	48.8	32	24.8
TT	32	50.0	25	39.1^1^	14	21.2
rs7218589						
CC	117	54.4	107	49.8	53	24.7
CG/GG	24	54.6	22	50.0	8	18.2
rs8068430						
TT	91	50.3	83	45.9	39	21.6
TC/CC	50	56.8	46	59.0	22	28.2
rs9807014						
CC	98	55.7	88	50.0	43	24.4
CT/TT	43	51.8	41	49.4	18	21.7

1 χ^2^ *P* < 0.05 for difference in seropositivity across genotypes.

2 Genus betaHPV species described in Table 2.

## Discussion

We measured cutaneous HPV and HPyV antibodies in samples collected immediately prior to transplant, before the impact of transplant-associated immunosuppression medications that might allow viral reactivation, to determine if antibodies detected prior to transplant were associated with later SCSC development. We found that a species 2 betaHPV type, HPV37, was significantly associated with a twofold increased risk of SCSC after transplant. In addition, infection with species 2 betaHPV was associated with 3 TMC6/8 variants among controls.

We also examined the risk of SCSC associated with six HPyV, as MCPyV DNA has been detected in SCSC tumor tissue in some 18,29 but not all studies 19,30. The lack of association with HPyV and most HPV in this study may indicate that unincluded or undiscovered cutaneous viruses may be associated with SCSC. Future studies may include previously unexamined cutaneous viruses to assess their influence on risk of SCSC.

Mucosal HPVs, specifically HPV16 and other high-risk types, have been recognized as a necessary causal factor in the majority of cervical, anogenital, and oropharyngeal cancers 31. They are mechanistically central to the development and maintenance of HPV-related cancers. In contrast, betaHPV may be involved in the development but not maintenance of malignancy. For example, cutaneous HPVs are often absent or present in very low copy numbers in SCSC tumors but present at higher levels in precancers 32. The E6 and E7 genes of some betaHPV types do appear to disrupt the p53 and pRb pathways in various ways, but less effectively than high-risk HPVs 33–36. In addition, betaHPVs may interact with UV-damaged cells to induce SCSC by interfering with DNA repair 37–40, cell cycle control 41,42, or increasing cell life span 39. The betaHPVs may act as promoters of SCSC after UV damage through a combination of these pathways, and genetic damage from UV exposure and betaPV infections are acquired years before transplant.

We observed an increased risk of SCSC associated with antibodies to HPV1, which is associated with plantar warts in the general population 43. This finding may suggest that transplant recipients who develop SCSC have a generalized susceptibility to some types of cutaneous HPV lesions. However, there was no increased risk of SCSC associated with antibodies to the two other cutaneous wart-causing cutaneous types (HPV2 and HPV4) measured.

Two prior case–control studies in transplant recipients 13,21 have reported on the relationship between serologic antibodies to HPV and risk of SCSC. Casabonne et al. 21. enrolled 140 cases and 454 controls from two British transplant centers and reported no associations between HPV seropositivity and SCSC risk, but did not include antigens for HPV37. Proby et al. 13. included 210 cases and 394 control transplant recipients from multiple centers across Europe. They found borderline statistically significant associations between risk of SCSC and a combined measure of antibody/hair follicle HPV DNA positivity for three types (HPV5 OR 2.0, 95% CI 0.95–4.3; HPV24 OR 2.0, 95% CI 0.9–4.1; HPV36 OR 2.4, 95% CI 1.0–5.4). Our study did not confirm an association with HPV24 (HPV24 OR 1.0, 95% CI 0.5–1.8), but did confirm the marginal association with HPV36 (OR 1.6, 95% CI 0.9-2.8). Proby et al. 13. did report an increased prevalence HPV37 DNA in plucked eyebrow hairs of cases compared to controls; however, HPV37 antigens were not included in their serologic analysis. Thus, HPV36 and HPV37 were associated with SCSC in transplant recipients in both studies.

Other studies have reported on HPV serology and SCSC risk in immune-competent populations 44–49. One found no association between HPV serology and risk of SCSC in a Swedish population 46, and another found an association with seropositivity to any HPV type among participants less than 50 years old 49. Another recent European study reported on risk of recurrence of SCSC was associated with seropositivity to HPV15, 24, 60, and 95 50. Andersson et al. 51 conducted a nested case–control study of 633 matched pairs, and found an increased risk of SCSC associated with species 2 betaHPV types (OR 1.3, 95% CI 1.1–1.7). Although HPV37 is a member of species 2, it was not included in their study; this study supports the suggestion for a role for species 2 betaHPV in SCSC.

Ramoz et al. found 11 that individuals with a rare dermatologic condition, epidermodysplasia verruciformis (EV), have a high risk of warts and SCSC associated with mutations in *TMC6*/*8*. This mutation may increase susceptibility to HPV infection, particularly betaHPVs. Karagas et al. reported SCSC risk in a US study was associated with multiple cutaneous HPV types 48. A subsequent study in the same population 52 found that a coding variant in *TMC8*, rs7208422, was associated with SCSC and betaHPV seropositivity among controls. This study found that rs7208422 was associated with seropositivity to species 2 betaHPV types among controls (Table 3). We also found that two other SNPs in TMC8 (rs12452890 and rs412611) were associated with seropositivity to species 2 betaHPVs. These findings add to the evidence that TMC mutations may influence the risk of species 2 betaHPV infections.

The study has several limitations and strengths. It is potentially limited by its sample size, which may miss modest associations. Another point to consider is that the controls were matched closely to cases, as described in the Materials and Methods section. Given that the prevalence of SCSC is high in this population, this close matching may have attenuated the effect estimates; however, matching might also be considered a strength of the study as immune response is likely a very important component of excess risk of SCSC in this population. A further limitation is that tumor virus status remains unknown, which would be a more definitive indication of a potential association between HPV37 and SCSC. Also, multiple comparisons are a potential source of false-positive results, but suggestive findings concerning species 2 antibodies and SCSC in other studies support the HPV37 finding. A strength of the study is that it represents one geographic area, and therefore a homogenous group. Although the reproducibility of the antibody assay was good, the results may not accurately reflect prevalence of prior exposure to specific HPV types measured. For example, the low proportion of samples found to be seropositive for HPV38 was likely an underestimate based on prior literature in transplant recipients 13,21,53. The nested case–control study approach and use of blood samples drawn before transplant make it unlikely that the exposure assessment was affected by immune suppression following transplant.

In summary, it is likely that relevant cutaneous betaHPV infections are acquired early in life and therefore prior to transplant, as demonstrated in family studies of betaHPV 54. Specific variants in the TMC6/8 gene region may contribute to susceptibility to species 2 infections. If transplant recipients have latent cutaneous HPV infections that are re-activated in the posttransplant period, the infections may promote cell growth, increasing the likelihood that UV-damaged cells acquire additional genetic damage. With respect to HPV37, the increased risk remained significant after correction for multiple comparisons and warrants further study. No clinical role for this finding is warranted at this time. HPV37 was originally isolated from a subtype of SCSC, a keratoacanthoma 55, suggesting that it may also be found in other SCSC tumors. A prospective study design with repeated measures before and after immunosuppression is the next step toward testing the putative cutaneous virus-SCSC association.

## Supporting Information

**Table S1**. Characteristics of SCOT Cohort study participants by matching variables.

**Table S2**. Risk of SCSC among transplant recipients related to *TMC6/8* variants.

## References

[1] Engels EA, Pfeiffer RM, Kasiske JF, Fraumeni BL, Israni AK, Snyder JJ (2011). Spectrum of cancer risk among US solid organ transplant recipients. JAMA.

[2] Euvrard S, Kanitakis J, Claudy A (2003). Skin cancers after organ transplantation. N. Engl. J. Med.

[3] Bouwes Bavinck JN, De BA, Vermeer BJ, Hartevelt MM, van der Woude FJ, Claas FH (1993). Sunlight, keratotic skin lesions and skin cancer in renal transplant recipients. Br. J. Dermatol.

[4] Jensen P, Hansen S, Moller B, Leivestad T, Pfeffer P, Geiran O (1999). Skin cancer in kidney and heart transplant recipients and different long-term immunosuppressive therapy regimens. J. Am. Acad. Dermatol.

[5] Lindelof B, Sigurgeirsson B, Gabel H, Stern RS (2000). Incidence of skin cancer in 5356 patients following organ transplantation. Br. J. Dermatol.

[6] Arron ST, Ruby JG, Dybbro E, Ganem D, Derisi JL (2011). Transcriptome sequencing demonstrates that human papillomavirus is not active in cutaneous squamous cell carcinoma. J. Invest. Dermatol.

[7] Berg D, Otley CC (2002). Skin cancer in organ transplant recipients: epidemiology, pathogenesis, and management. J. Am. Acad. Dermatol.

[8] Euvrard S, Kanitakis J, Decullier E, Butnaru AC, Lefrancois N, Boissonnat P (2006). Subsequent skin cancers in kidney and heart transplant recipients after the first squamous cell carcinoma. Transplantation.

[9] Orth G (2005). Human papillomaviruses associated with epidermodysplasia verruciformis in non-melanoma skin cancers: guilty or innocent?. J. Invest. Dermatol.

[10] Pfister H (2003). Chapter 8: human papillomavirus and skin cancer. J. Natl. Cancer Inst. Monogr.

[11] Ramoz N, Taieb A, Rueda LA, Montoya LS, Bouadjar B, Favre M (2000). Evidence for a nonallelic heterogeneity of epidermodysplasia verruciformis with two susceptibility loci mapped to chromosome regions 2p21-p24 and 17q25. J. Invest. Dermatol.

[12] Lazarczyk M, Cassonnet P, Pons C, Jacob Y, Favre M (2009). The EVER proteins as a natural barrier against papillomaviruses: a new insight into the pathogenesis of human papillomavirus infections. Microbiol. Mol. Biol. Rev.

[13] Proby CM, Harwood CA, Neale RE, Green AC, Euvrard S, Naldi L (2011). A case-control study of betapapillomavirus infection and cutaneous squamous cell carcinoma in organ transplant recipients. Am. J. Transplant.

[14] Harwood CA, Surentheran T, McGregor JM, Spink PJ, Leigh IM, Breuer J (2000). Human papillomavirus infection and non-melanoma skin cancer in immunosuppressed and immunocompetent individuals. J. Med. Virol.

[15] Reuschenbach M, Tran T, Faulstich F, Hartschuh W, Vinokurova S, Kloor M (2011). High-risk human papillomavirus in non-melanoma skin lesions from renal allograft recipients and immunocompetent patients. Br. J. Cancer.

[16] Feng H, Shuda M, Chang Y, Moore PS (2008). Clonal integration of a polyomavirus in human Merkel cell carcinoma. Science.

[17] Sihto H, Kukko H, Koljonen V, Sankila R, Bohling T, Joensuu H (2009). Clinical factors associated with Merkel cell polyomavirus infection in Merkel cell carcinoma. J. Natl. Cancer Inst.

[18] Rollison DE, Giuliano AR, Messina JL, Fenske NA, Cherpelis BS, Sondak VK (2012). Case-control study of Merkel cell polyomavirus infection and cutaneous squamous cell carcinoma. Cancer Epidemiol. Biomarkers Prev.

[19] Ridd K, Yu S, Bastian BC (2009). The presence of polyomavirus in non-melanoma skin cancer in organ transplant recipients is rare. J. Invest. Dermatol.

[20] Moens U, Ludvigsen M, Van GM (2011). Human polyomaviruses in skin diseases. Patholog. Res. Int.

[21] Casabonne D, Lally A, Mitchell L, Michael KM, Waterboer T, Pawlita M (2009). A case-control study of cutaneous squamous cell carcinoma among Caucasian organ transplant recipients: the role of antibodies against human papillomavirus and other risk factors. Int. J. Cancer.

[22] Madeleine MM, Johnson LG, Daling JR, Schwartz SM, Carter JJ, Berg D (2013). Cohort profile: the skin cancer after organ transplant study. Int. J. Epidemiol.

[23] Sehr P, Muller M, Hopfl R, Widschwendter A, Pawlita M (2002). HPV antibody detection by ELISA with capsid protein L1 fused to glutathione S-transferase. J. Virol. Methods.

[24] Waterboer T, Sehr P, Michael KM, Franceschi S, Nieland JD, Joos TO (2005). Multiplex human papillomavirus serology based on in situ-purified glutathione s-transferase fusion proteins. Clin. Chem.

[25] Orozco JJ, Carter JJ, Koutsky LA, Galloway DA (2005). Humoral immune response recognizes a complex set of epitopes on human papillomavirus type 6 L1 capsomers. J. Virol.

[26] Carter JJ, Paulson KG, Wipf GC, Miranda D, Madeleine MM, Johnson LG (2009). Association of Merkel cell polyomavirus-specific antibodies with Merkel cell carcinoma. J. Natl. Cancer Inst.

[27] Edlund CK, Lee WH, Li D, Van Den Berg DJ, Conti DV (2008). Snagger: a user-friendly program for incorporating additional information for tagSNP selection. BMC Bioinformatics.

[28] Ludbrook J (1998). Multiple comparison procedures updated. Clin. Exp. Pharmacol. Physiol.

[29] Dworkin AM, Tseng SY, Allain DC, Iwenofu OH, Peters SB, Toland AE (2009). Merkel cell polyomavirus in cutaneous squamous cell carcinoma of immunocompetent individuals. J. Invest. Dermatol.

[30] Reisinger DM, Shiffer JD, Chang AB, Cognetta Y, Moore PS (2010). Lack of evidence for basal or squamous cell carcinoma infection with Merkel cell polyomavirus in immunocompetent patients with Merkel cell carcinoma. J. Am. Acad. Dermatol.

[31] zur Hausen H (2000). Papillomaviruses causing cancer: evasion from host-cell control in early events in carcinogenesis. J. Natl. Cancer Inst.

[32] Weissenborn SJ, Nindl I, Purdie K, Harwood C, Proby C, Breuer J (2005). Human papillomavirus-DNA loads in actinic keratoses exceed those in non-melanoma skin cancers. J. Invest. Dermatol.

[33] Cornet I, Bouvard V, Campo MS, Thomas M, Banks L, Gissmann L (2012). Comparative analysis of transforming properties of E6 and E7 from different beta human papillomavirus types. J. Virol.

[34] Caldeira S, Zehbe I, Accardi R, Malanchi I, Dong W, Giarre M (2003). The E6 and E7 proteins of the cutaneous human papillomavirus type 38 display transforming properties. J. Virol.

[35] Accardi R, Dong W, Smet A, Cui R, Hautefeuille A, Gabet AS (2006). Skin human papillomavirus type 38 alters p53 functions by accumulation of deltaNp73. EMBO Rep.

[36] Howie HL, Koop JI, Weese J, Robinson K, Wipf G, Kim L (2011). Beta-HPV 5 and 8 E6 promote p300 degradation by blocking AKT/p300 association. PLoS Pathog.

[37] Giampieri S, Storey A (2004). Repair of UV-induced thymine dimers is compromised in cells expressing the E6 protein from human papillomaviruses types 5 and 18. Br. J. Cancer.

[38] Struijk L, van der Meijden E, Kazem S, Ter SJ, de Gruijl FR, Steenbergen RD (2008). Specific betapapillomaviruses associated with squamous cell carcinoma of the skin inhibit UVB-induced apoptosis of primary human keratinocytes. J. Gen. Virol.

[39] Underbrink MP, Howie HL, Bedard KM, Koop JI, Galloway DA (2008). E6 proteins from multiple human betapapillomavirus types degrade Bak and protect keratinocytes from apoptosis after UVB irradiation. J. Virol.

[40] Muschik D, Braspenning-Wesch I, Stockfleth E, Rosl F, Hofmann TG, Nindl I (2011). Cutaneous HPV23 E6 prevents p53 phosphorylation through interaction with HIPK2. PLoS ONE.

[41] Giampieri S, Garcia-Escudero R, Green J, Storey A (2004). Human papillomavirus type 77 E6 protein selectively inhibits p53-dependent transcription of proapoptotic genes following UV-B irradiation. Oncogene.

[42] Viarisio D, Mueller-Decker K, Kloz U, Aengeneyndt B, Kopp-Schneider A, Grone HJ (2011). E6 and E7 from beta HPV38 cooperate with ultraviolet light in the development of actinic keratosis-like lesions and squamous cell carcinoma in mice. PLoS Pathog.

[43] Chow LT, Reilly SS, Broker TR, Taichman LB (1987). Identification and mapping of human papillomavirus type 1 RNA transcripts recovered from plantar warts and infected epithelial cell cultures. J. Virol.

[44] de Koning M, Quint W, Struijk L, Kleter B, Wanningen P, van Doorn LJ (2006). Evaluation of a novel highly sensitive, broad-spectrum PCR-reverse hybridization assay for detection and identification of beta-papillomavirus DNA. J. Clin. Microbiol.

[45] Waterboer T, Abeni D, Sampogna F, Rother A, Masini C, Sehr P (2008). Serological association of beta and gamma human papillomaviruses with squamous cell carcinoma of the skin. Br. J. Dermatol.

[46] Andersson K, Waterboer T, Kirnbauer R, Slupetzky K, Iftner T, de Villiers EM (2008). Seroreactivity to cutaneous human papillomaviruses among patients with nonmelanoma skin cancer or benign skin lesions. Cancer Epidemiol. Biomarkers Prev.

[47] Bouwes Bavinck JN, Neale RE, Abeni D, Euvrard S, Green AC, Harwood CA (2010). Multicenter study of the association between betapapillomavirus infection and cutaneous squamous cell carcinoma. Cancer Res.

[48] Karagas MR, Waterboer T, Li Z, Nelson HH, Michael KM, Bavinck JN (2010). Genus beta human papillomaviruses and incidence of basal cell and squamous cell carcinomas of skin: population based case-control study. BMJ.

[49] Plasmeijer EI, Pandeya N, O'Rourke P, Pawlita M, Waterboer T, Feltkamp MC (2011). The Association between cutaneous squamous cell carcinoma and betapapillomavirus seropositivity: a cohort study. Cancer Epidemiol. Biomarkers Prev.

[50] Paradisi A, Waterboer T, Sampogna F, Tabolli S, Simoni S, Pawlita M (2011). Seropositivity for human papillomavirus and incidence of subsequent squamous cell and basal cell carcinomas of the skin in patients with a previous nonmelanoma skin cancer. Br. J. Dermatol.

[51] Andersson K, Michael KM, Luostarinen T, Waterboer T, Gislefoss R, Hakulinen T (2012). Prospective study of human papillomavirus seropositivity and risk of nonmelanoma skin cancer. Am. J. Epidemiol.

[52] Patel AS, Karagas MR, Pawlita M, Waterboer T, Nelson HH (2008). Cutaneous human papillomavirus infection, the EVER2 gene and incidence of squamous cell carcinoma: a case-control study. Int. J. Cancer.

[53] Sampogna F, Bavinck JN, Pawlita M, Abeni D, Harwood CA, Proby CM (2012). Factors associated with the seroprevalence of 26 cutaneous and two genital human papillomavirus types in organ transplant patients. J. Gen. Virol.

[54] Weissenborn SJ, de Koning MN, Wieland U, Quint WG, Pfister HJ (2009). Intrafamilial transmission and family-specific spectra of cutaneous betapapillomaviruses. J. Virol.

[55] Scheurlen W, Gissmann L, Gross G, zur Hausen H (1986). Molecular cloning of two new HPV types (HPV 37 and HPV 38) from a keratoacanthoma and a malignant melanoma. Int. J. Cancer.

[56] de Villiers EM, Fauquet C, Broker TR, Bernard HU, Zur HH (2004). Classification of papillomaviruses. Virology.

