# Supplementary material for: Risk of squamous cell skin cancer after organ transplant associated with antibodies to cutaneous papillomaviruses, polyomaviruses, and TMC6/8 (EVER1/2) variants
Source: Cancer Med. 2014 Jun 10;3(5):1440–7. doi: 10.1002/cam4.280 (PMC4302694; doi:10.1002/cam4.280)
Supplement: Supplementary file 1 — Table S1. Characteristics of SCOT Cohort study participants by matching variables. [file cam40003-1440-SD1.docx]

Supplemental Table 1. Characteristics of SCOT Cohort study participants by matching variables

|  | **Controls** | | **Cases** | |
| --- | --- | --- | --- | --- |
|  | **(N=329)** | | **(N=172)** | |
|  | **n** | **(%)** | **n** | **(%)** |
|  |  |  |  |  |
| Year of transplant |  |  |  |  |
| 1995-99 | 97 | 29.5 | 65 | 37.8 |
| 2000-04 | 149 | 45.3 | 71 | 41.3 |
| 2005-10 | 83 | 25.2 | 36 | 20.9 |
| Age at transplant |  |  |  |  |
| 18-49 | 103 | 31.3 | 42 | 24.4 |
| 50-64 | 177 | 53.8 | 100 | 58.1 |
| 65+ | 49 | 14.9 | 30 | 17.4 |
| Gender |  |  |  |  |
| Male | 250 | 76.0 | 132 | 76.7 |
| Female | 79 | 24.0 | 40 | 23.3 |
| Race |  |  |  |  |
| White | 292 | 88.8 | 164 | 95.3 |
| Non-white | 37 | 11.2 | 8 | 4.7 |
| Time since transplant |  |  |  |  |
| < 2 years | 43 | 13.1 | 22 | 12.8 |
| 2 to < 5 years | 133 | 40.4 | 69 | 40.1 |
| 5 to < 10 years | 122 | 37.1 | 63 | 36.6 |
| 10+ years | 31 | 9.4 | 18 | 10.5 |
| Hospital |  |  |  |  |
| Swedish | 66 | 20.1 | 42 | 24.4 |
| University of Washington | 172 | 52.3 | 85 | 49.4 |
| Virginia Mason | 91 | 27.7 | 44 | 25.6 |
| Age at diagnosis/reference |  |  |  |  |
| 18-49 | 44 | 13.4 | 19 | 11.0 |
| 50-64 | 201 | 61.1 | 87 | 50.6 |
| 65+ | 84 | 25.5 | 66 | 38.4 |
| Organ transplanted |  |  |  |  |
| Kidney | 269 | 81.8 | 137 | 79.7 |
| Heart | 60 | 18.2 | 35 | 20.3 |
| Kidney donor type |  |  |  |  |
| Deceased | 164 | 61.2 | 77 | 56.2 |
| Living related | 57 | 21.3 | 32 | 23.4 |
| Living unrelated | 44 | 16.4 | 27 | 19.7 |
| Unknown/missing | 3 | 1.1 | 1 | 0.7 |
