# Supplementary material for: Risk of squamous cell skin cancer after organ transplant associated with antibodies to cutaneous papillomaviruses, polyomaviruses, and TMC6/8 (EVER1/2) variants
Source: Cancer Med. 2014 Jun 10;3(5):1440–7. doi: 10.1002/cam4.280 (PMC4302694; doi:10.1002/cam4.280)
Supplement: Supplementary file 2 — Table S2. Risk of SCSC among transplant recipients related to TMC6/8 variants. [file cam40003-1440-SD2.docx]

Supplementary Table 2. Risk of SCSC among transplant recipients related to *TMC6/8* variants

|  | Controls | | Cases | | Log- |  |
| --- | --- | --- | --- | --- | --- | --- |
|  | (n=282) | | (n=156) | | additive |  |
| Variant | n | % | n | % | OR^1^ | (95% CI) |
| rs12452890 | | | |  | 0.92 | (0.67-1.27) |
| AA | 61 | (23.6) | 36 | (25.2) |  |  |
| AG | 134 | (51.7) | 77 | (53.8) |  |  |
| GG | 64 | (24.7) | 30 | (21.0) |  |  |
| rs16970842^2^ | | | | | 0.56 | (0.29-1.08) |
| AA | 210 | (81.1) | 125 | (87.4) |  |  |
| AG | 48 | (18.5) | 18 | (12.6) |  |  |
| GG | 1 | (0.4) | 0 | (0.0) |  |  |
| rs16970849 | | | |  | 0.73 | (0.31-1.69) |
| GG | 237 | (91.5) | 133 | (93.0) |  |  |
| GA | 21 | (8.1) | 9 | (6.3) |  |  |
| AA | 1 | (0.4) | 1 | (0.7) |  |  |
| rs2871647^2^ | | | |  | 1.35 | (0.78-2.33) |
| AA | 213 | (82.2) | 112 | (78.3) |  |  |
| AC | 45 | (17.4) | 31 | (21.7) |  |  |
| CC | 1 | (0.4) | 0 | (0.0) |  |  |
| rs383603 | | | |  | 0.84 | (0.58-1.20) |
| CC | 153 | (59.1) | 91 | (63.6) |  |  |
| CG | 89 | (34.4) | 45 | (31.5) |  |  |
| GG | 17 | (6.6) | 7 | (4.9) |  |  |
| rs412611^2^ | | | |  | 0.78 | (0.40-1.50) |
| GG | 222 | (85.7) | 128 | (89.5) |  |  |
| GA | 37 | (14.3) | 15 | (10.5) |  |  |
| rs454138 | | | |  | 0.92 | (0.66-1.27) |
| GG | 95 | (36.7) | 54 | (37.8) |  |  |
| GC | 122 | (47.1) | 71 | (49.7) |  |  |
| CC | 42 | (16.2) | 18 | (12.6) |  |  |
| rs7208422 | | | |  | 0.87 | (0.68-1.10) |
| AA | 66 | (25.5) | 34 | (23.8) |  |  |
| AT | 129 | (49.8) | 81 | (56.6) |  |  |
| TT | 64 | (24.7) | 28 | (19.6) |  |  |
| rs7218589^2^ | | | |  | 0.53 | (0.27-1.04) |
| CC | 215 | (83.0) | 128 | (89.5) |  |  |
| CG | 44 | (17.0) | 14 | (9.8) |  |  |
| GG | 0 | (0.0) | 1 | (0.7) |  |  |
| rs8068430^2^ | | | |  | 1.28 | (0.80-2.05) |
| TT | 181 | (69.9) | 92 | (64.3) |  |  |
| TC | 72 | (27.8) | 48 | (33.6) |  |  |
| CC | 6 | (2.3) | 3 | (2.1) |  |  |
| rs9807014 | | | | | 1.18 | (0.78-1.77) |
| CC | 176 | (68.0) | 97 | (67.8) |  |  |
| CT | 77 | (29.7) | 40 | (28.0) |  |  |
| TT | 6 | (2.3) | 6 | (4.2) |  |  |

^1^Odds ratios (OR).

^2^Genotypes modeled as dominant.
